# Supplementary material for: Aryl Hydrocarbon Receptor Repressor Is Hypomethylated in Psoriasis and Promotes Psoriasis-like Inflammation in HaCaT Cells
Source: Int J Mol Sci. 2021 Nov 24;22(23):12715. doi: 10.3390/ijms222312715 (PMC8657998; doi:10.3390/ijms222312715)
Supplement: Supplementary file 1 [file ijms-22-12715-s001.zip › ijms-1441363-supplementary.pdf]

Supplement figures legends

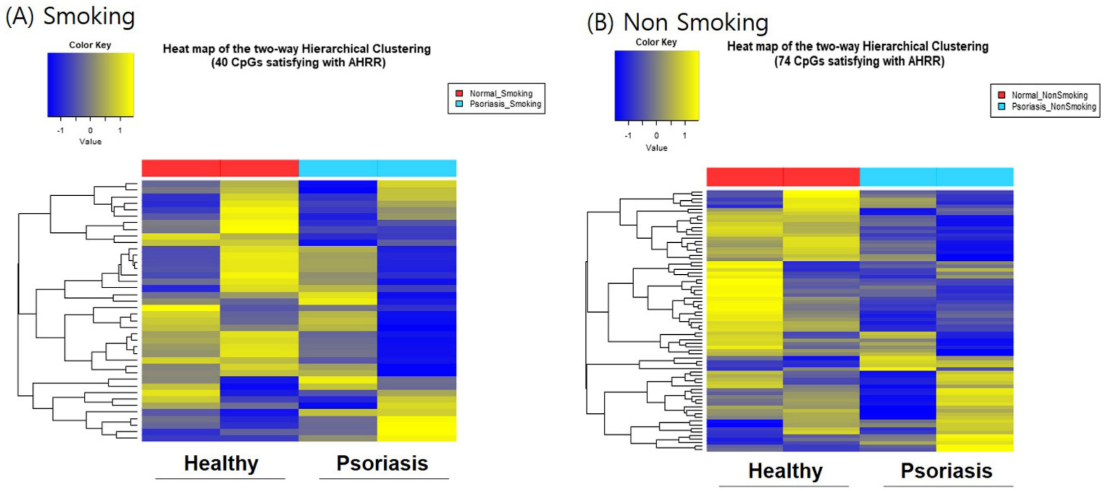

Figure S1. Effect of *AhRR* hypomethylation in PBMC of psoriasis patients

Data corresponding to the *AhRR* gene of healthy and psoriasis samples are displayed as a color-coded gene-by-sample heatmap, with rows (genes) and columns (samples) sorted by hierarchical clustering. Blue is hypomethylation and yellow is hypermethylation. DNA methylation from peripheral blood mononuclear cells (PBMC) of psoriasis patients and PBMC of healthy volunteers exhibited hypomethylation in several loci of the *AhRR* gene in patients with psoriasis. We considered cigarette smoke as one of the common environmental factors and divided them into normal and psoriasis patients with (Supplement 1A) or without smoking (Supplement 1B).

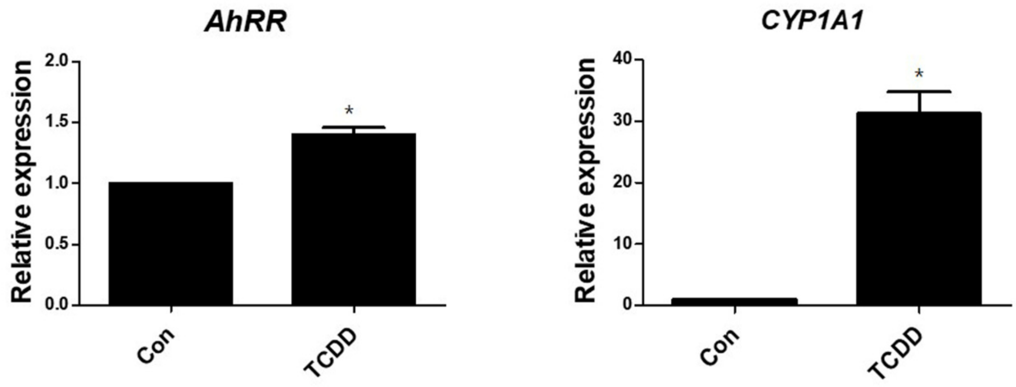

Figure S2. Effect of TCDD on inflammatory cytokine in Normal Human_Epidermal Keratinocytes (NHEK).

NHEK were treated with TCDD (500 nM) for 24 h. *AhRR*, *CYP1A1* mRNA expression were examined by qPCR. Values are means ± SEM. All experiments of at least three independent experiments. *$p < 0.05$ vs. control. To investigate the effect of TCDD on AhRR expression, NHEK were treated with TCDD and *AhRR* and *CYP1A1* expression were determined. As a result, expression of *AhRR* and *CYP1A1* levels significantly increased in following TCDD treatment.
